# Fungus *Metarhizium robertsii* and neurotoxic insecticide affect gut immunity and microbiota in Colorado potato beetles

**DOI:** 10.1038/s41598-020-80565-x

**Published:** 2021-01-14

**Authors:** Vadim Yu. Kryukov, Ulyana Rotskaya, Olga Yaroslavtseva, Olga Polenogova, Natalia Kryukova, Yuriy Akhanaev, Anton Krivopalov, Tatyana Alikina, Yana L. Vorontsova, Irina Slepneva, Marsel Kabilov, Viktor V. Glupov

**Affiliations:** 1 Institute of Systematics and Ecology of Animals, Siberian Branch of Russian Academy of Sciences, Novosibirsk, 630091 Russia; 2 Tomsk State University, Tomsk, 634050 Russia; 3 Institute of Chemical Biology and Fundamental Medicine, Siberian Branch of Russian Academy of Sciences, Novosibirsk, 630090 Russia; 4 Voevodsky Institute of Chemical Kinetics and Combustion, Siberian Branch of Russian Academy of Sciences, Novosibirsk, 630090 Russia

**Keywords:** Psychology, Microbial communities, Pathogens, Microbiology, Fungi, Fungal host response

## Abstract

Fungal infections and toxicoses caused by insecticides may alter microbial communities and immune responses in the insect gut. We investigated the effects of *Metarhizium robertsii* fungus and avermectins on the midgut physiology of Colorado potato beetle larvae. We analyzed changes in the bacterial community, immunity- and stress-related gene expression, reactive oxygen species (ROS) production, and detoxification enzyme activity in response to topical infection with the *M. robertsii* fungus, oral administration of avermectins*,* and a combination of the two treatments. Avermectin treatment led to a reduction in microbiota diversity and an enhancement in the abundance of enterobacteria, and these changes were followed by the downregulation of *Stat* and *Hsp*90, upregulation of transcription factors for the Toll and IMD pathways and activation of detoxification enzymes. Fungal infection also led to a decrease in microbiota diversity, although the changes in community structure were not significant, except for the enhancement of *Serratia*. Fungal infection decreased the production of ROS but did not affect the gene expression of the immune pathways. In the combined treatment, fungal infection inhibited the activation of detoxification enzymes and prevented the downregulation of the JAK-STAT pathway caused by avermectins. The results of this study suggest that fungal infection modulates physiological responses to avermectins and that fungal infection may increase avermectin toxicosis by blocking detoxification enzymes in the gut.

## Introduction

Insect microbial associates play important roles in the development of diseases caused by entomopathogens^1^ and toxicoses caused by insecticides^2^. Different types of interactions may be observed between bacterial symbionts and insect pathogenic ascomycetes (e.g., *Beauveria* and *Metarhizium*), which infect their hosts primarily through integuments and, less frequently, through the gut. These fungi may interact with host bacteria directly on the cuticle surface or in the gut. In these cases, bacteria inhibit fungal growth and differentiation, thereby acting as a component of the defense system against fungal infections^3–6^. In contrast, indirect interactions between fungi and symbiotic bacteria may be mediated by host immune responses. These more complicated interactions include the spatial relocation of physiological reactions, which may lead to synergy between the microorganisms and accelerated mortality. For example, topical infection of the mosquito *Anopheles* with the fungus *Beauveria bassiana* led to the proliferation of bacteria *Serratia marcescens* in the gut and hemocoel, thereby promoting fungal infection^7^. Similar effects were observed for the bark beetle *Dendroctonus valens*^8^ and the wax moth *Galleria mellonella*^9^.

In natural and agricultural habitats, insects are exposed to a broad range of entomopathogenic microorganisms and various toxicants, such as plant secondary metabolites, synthetic and natural insecticides, and parasitoid venoms. It is well-known that these toxicants may significantly modulate the susceptibility of hosts to fungal pathogens^10,11^. Various approaches for integrated pest control have been developed on this basis^12–18^. Researchers usually explain synergy between pathogenic fungi and insecticides by altering the immune response to fungal infections, as well as developmental and behavioral disorders. Importantly, xenobiotics may lead to changes in microbial communities in different ways. For example, certain insecticides cause different dysfunctions in the gut, such as alterations in peristaltic intensity, pH, and gut tissue damage^19^. Moreover, different toxicants may affect the immune and stress reactions in the gut. These effects may lead to changes in the bacterial community and, consequently, to changes in the susceptibility to fungi^9,20^.

Homeostasis in the gut bacterial community is determined by immune-physiological reactions, with many of these reactions producing reactive oxygen species (ROS) and antimicrobial peptides (AMPs)^11,21^. The level of ROS is dictated by the Mesh-DUOX system^22^. It was proposed that DUOX generates superoxides outside the cells, which are unstable and are rapidly converted to H_2_O_2_^23^. In addition, the DUOX peroxidase homology domain may serve to convert H_2_O_2_ to HOCl^24^. All of these compounds are powerful oxidants and exhibit microbicidal activity^24^. It was shown for *Drosophila* and mosquito species that the Mesh-DUOX system is essential for both resistance to pathogens and the management of commensal microbial populations^22,24–26^. The inhibition of the Mesh-DUOX system leads to uncontrolled proliferation of bacteria in the gut and a reduction in insect survival^7,22^. Interestingly, downregulation of DUOX in the mosquito gut was observed after topical infection with the entomopathogenic fungus *B. bassiana,* as shown by Wei et al.^7^. However, contradictory results were obtained by Ramirez et al.^27^. AMP production is controlled by three major immune-signaling pathways: Toll, IMD and JAK-STAT. The Toll pathway is involved in the defense against gram-positive bacteria and fungi, the IMD pathway is primarily involved in the defense against gram-negative bacteria, and the JAK-STAT pathway is involved in the defense against viruses but also against fungi^28^ and bacteria^26,29^. Changes in the expression levels of the transcription factors *Dif/Dorsal*, *Relish* and *Stat* (or their orthologs) indicate the activation/inhibition of the Toll, IMD and JAK-STAT pathways, respectively^27^. Topical fungal infection may both downregulate and upregulate the expression of these transcription factors and AMPs in the gut, depending on certain models of pathogenesis^7,9,27^.

In insects, stress responses may play important roles in the response to fungi, bacteria and insecticides. In particular, heat-shock proteins are activated under various stress conditions, including infections and toxicoses^30,31^. These proteins participate in the stabilization and transport of proteins and are therefore involved in cell repair, immune-signaling pathways and other vital processes^31,32^. Antioxidants and detoxification enzymes participate in the inactivation of insecticides and microbial metabolites in various organs and tissues of insects, including the gut^33^. Esterases (ESTs) function against a broad range of insecticides^34^, bacterial toxins^33,35^ and metabolites formed under mycoses^36^. Glutathione-*S*-transferases (GSTs) participate in ROS inactivation and are involved in the metabolism of insecticides from different classes^34^ and are likely involved in the metabolism of fungal toxins^37^. The abovementioned immune responses in the insect gut during the development of mycoses or toxicoses caused by insecticides have not been fully elucidated, particularly when the fungi and insecticides are combined.

The Colorado potato beetle *Leptinotarsa decemlineata* (Say) is an important pest in most potato-producing regions around the world. The rapid development of resistance to various chemical insecticides requires the development of integrated approaches to the control of *L. decemlineata* populations^38^. One of these approaches may be employing a combination of insecticides and entomopathogens^13,17,39,40^. Previously, we studied the immunophysiological mechanisms of the synergy between the fungus *Metarhizium robertsii* and the macrocyclic lactones avermectins after a combined treatment to control the Colorado potato beetle^39,40^. We have shown that avermectins cause a delay in larval development and inhibit cellular immunity, which disrupts the immune response against fungal infection. In this study, we attempted to determine how these agents influence the gut microbiota and gut immunity and whether these changes contribute to the synergy between the fungus and avermectins. We measured the changes in the microbiota structure, ROS production, detoxification system activity, and gene expression involved in the immune response and stress management in the Colorado potato beetle midgut under the oral administration of avermectins, topical infection with *M. robertsii* and combined treatment with these agents*.*

## Results

### Bioassay

**Figure 1:**
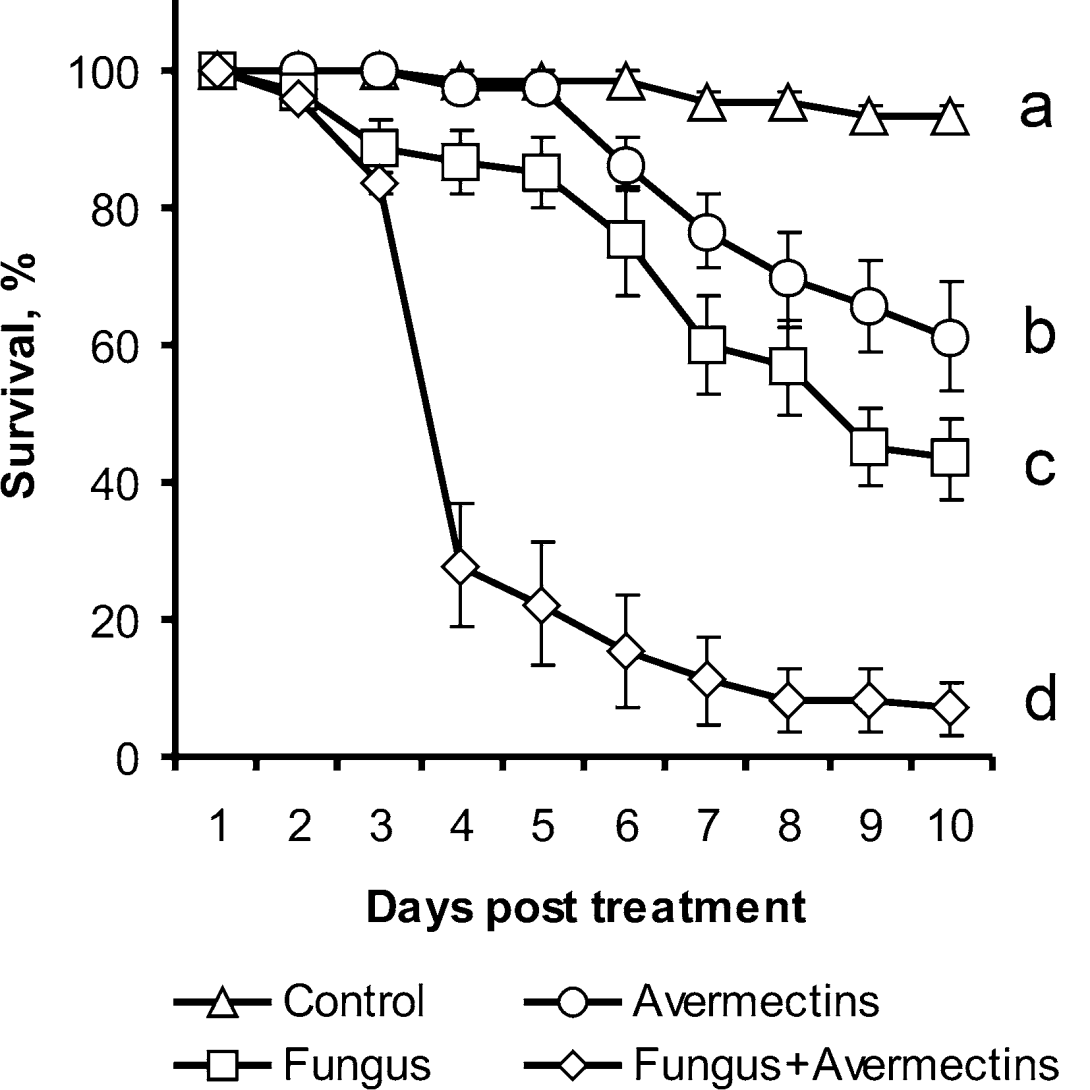
Survival of Colorado potato beetle larvae after topical treatment with *M. robertsii* conidia, oral administration of avermectins and the combined treatment. Different letters indicate significant differences in survival dynamics (log rank test, χ^2^ > 8.4, df = 1, P < 0.005). Synergy was observed from 4 to 10 days (χ^2^ > 10.2 df = 1, P < 0.001).

Treatment of Colorado potato beetle larvae with *M. robertsii*, avermectins and a combination of the two led to significant differences in the survival dynamics (log rank test: χ^2^ > 8.4, df = 1, P < 0.005, Fig. 1). Strong synergy between avermectin toxicosis and fungal infection in larval mortality was demonstrated from 4 to 10 days after treatment (χ^2^ > 10.2, df = 1, P < 0.001). At 10 days after treatment, the control larvae survived at a rate of 93.3%, whereas oral administration of avermectin and topical infection with fungus resulted in 61.1% and 43.3% survival, respectively, and the combined treatment resulted in 6.9% survival. Larvae that died after treatment with the fungus alone were primarily mummified (86%). Larvae that died after the combined treatment were mummified (39%) or decomposed by bacteria (61%), which was manifested by decay of cadavers with the appearance of an ammonia smell.

### Bacterial communities

An analysis of 16S rRNA gene sequencing in the whole experiment demonstrated the presence of 171 operative taxonomical units (OTUs) that belonged to bacteria from 63 families, 21 classes and 11 phyla (see Appendix 1). The predominant groups were enterobacteria (Proteobacteria, Enterobacteriaceae) and spiroplasmas (Tenericutes, Spiroplasmataceae). Blast analysis with GenBank-type material showed that the most abundant OTU, OTU-1, had 99.5–100% similarity with various species of *Enterobacter* and *Klebsiella*. The second most predominant OTU, OTU-20, showed high similarity (99.8%) only with *Citrobacter* strains. *Spiroplasma* (OTU-3) was identical (100%) only with *Spiroplasma leptinotarsae* (strain LD-1B), which is an obligate symbiont of the Colorado potato beetle^41^. In addition, relatively high abundances of OTUs were identified as *Serratia* (Enterobacteriaceae), *Acinetobacter* (Moraxellaceae), *Pseudomonas* (Pseudomonadaceae) and *Lactococcus* (Streptococcaceae).

**Figure 2:**
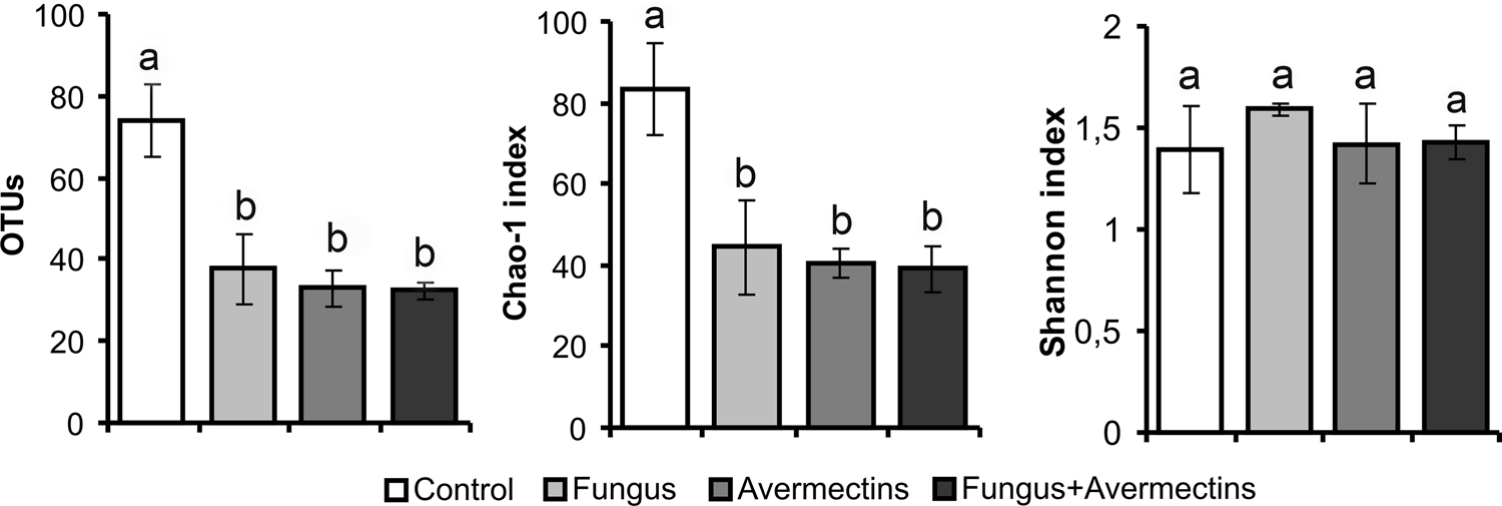
Diversity indexes of midgut bacterial communities of Colorado potato beetle larvae at 48 h after topical treatment with *M. robertsii* conidia, oral administration of avermectins and the combined treatment. Different letters indicate significant differences between treatments (Tukey’s test, P < 0.05).

Fungal infection and avermectin toxicosis led to a reduction in the diversity of the midgut bacterial community (Fig. 2). In particular, we registered a 2- to 2.4-fold decrease in the total OTU counts (effect of fungus—F_1,12_ = 10.4, P = 0.007; effect of avermectins—F_1,12_ = 16.5, P = 0.002), as well as a 1.9- to 2.2-fold decrease in the Chao-1 index (effect of fungus—F_1,12_ = 7.3, P = 0.02; effect of avermectins—F_1,12_ = 10.4, P = 0.007). Notably, the combined treatment did not lead to a stronger decrease in diversity compared to the single treatments (Tukey’s test, P > 0.89). Based on changes in the Shannon index, significant effects were not detected (F_1,12_ < 1.3, P > 0.28).

**Figure 3:**
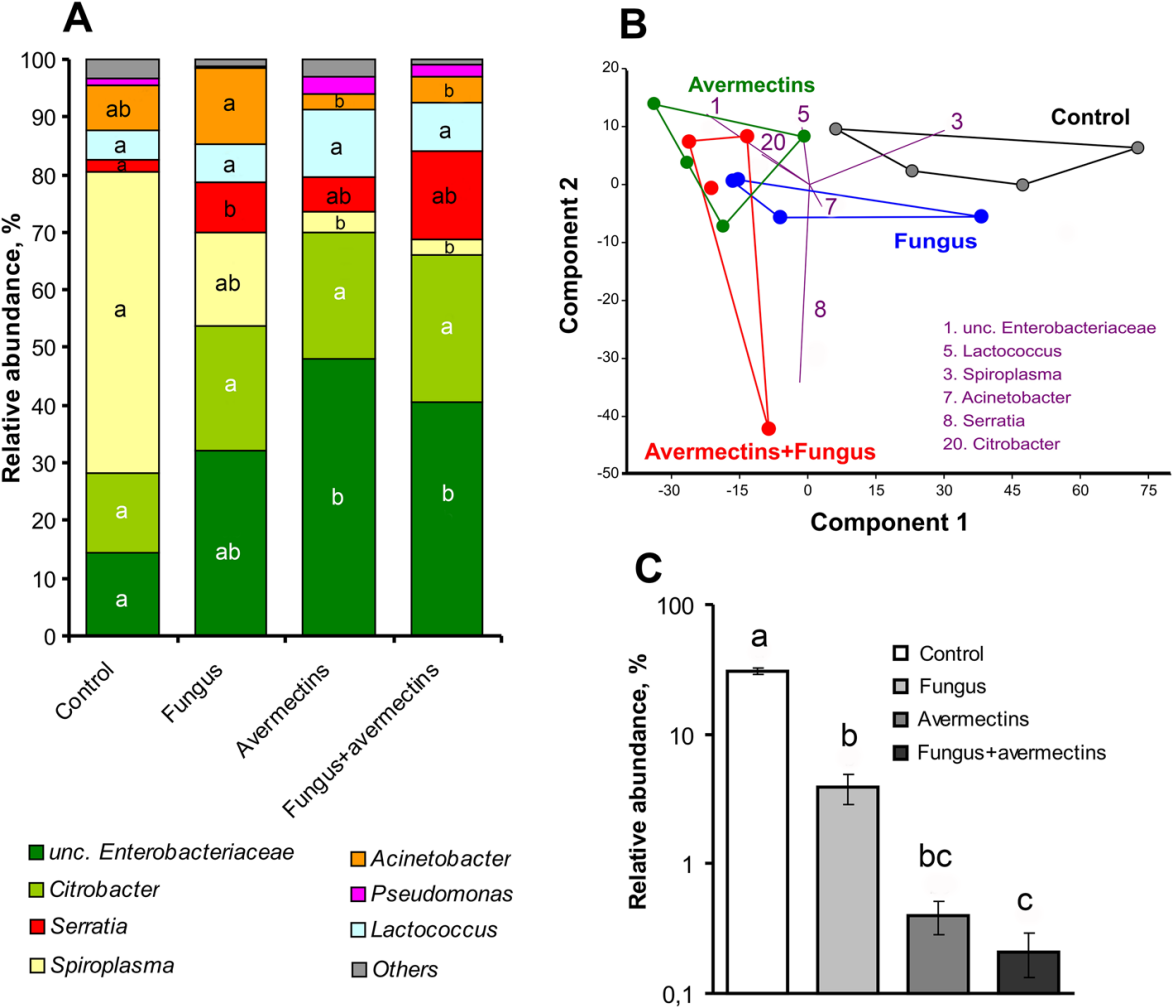
Alterations in bacterial communities in the midgut of Colorado potato beetle larvae at 48 h after topical treatment with *M. robertsii* conidia, oral administration of avermectins and the combined treatment. (**A**) Microbiota structure at the genus level, (**B**) PCA at the OTU level, with numbers indicating the predominant OTUs. (**C**) Changes in chloroplast 16S DNA relative abundance. Different letters indicate significant differences in taxa abundance between treatments (Dunn’s test, P < 0.05).

An analysis of the predominant taxa abundance showed that the effects of fungal infection were not significant, although trends of decreased abundance of *Spiroplasma* and increased abundance of unclassified Enterobacteriaceae and *Serratia* were observed (effect of fungus: H_1.15_ < 3.4, P > 0.059, Fig. 3A). However, fungal infection alone led to a significant increase in *Serratia* abundance compared to the control (Dunn’s test, P = 0.02). Avermectins caused a strong elevation of unclassified Enterobacteriaceae and decrease in *Spiroplasma* relative abundance (effect of avermectins: H_1,15_ > 5.3, P < 0.02). In addition, an increasing trend for *Pseudomonas* was observed after treatment with avermectins (H_1,15_ = 2.8, P = 0.09). Factor interactions (fungus × avermectins) in the changes of the predominant group of bacteria were not observed (H_1,15_ < 1.9, P > 0.17). The structure of bacterial communities was similar in the larvae treated with avermectins and the combination of avermectins and fungus. It is important to note that fungal infection and avermectin toxicosis led to a decrease in the relative abundance of chloroplast DNA by 10- and 100-fold, respectively (H_1,15_ > 11.0, P < 0.001, Fig. 3C), which indicates a disruption in the nutrition of treated insects.

A principal component analysis showed clear clustering between communities of control insects and avermectin-treated insects (Fig. 3B). The community of insects treated with fungus alone occupied an intermediate location. The first component explained 66.0% of the variation, which was primarily due to changes in *Spiroplasma* and Enterobacteriaceae abundance. The second component explained 13.0% of the variation caused by an increase in *Serratia* abundance under the condition of fungal infection.

### Changes in colony-forming unit (CFU) count

**Figure 4:**
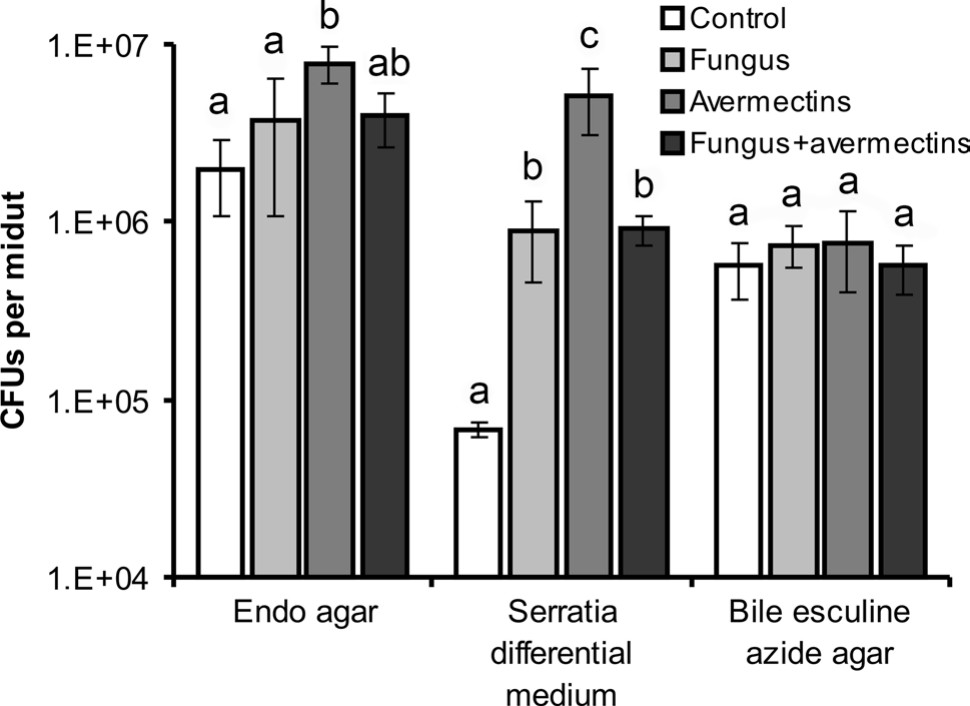
CFU count in the midgut of Colorado potato beetle larvae at 48 h after topical treatment with *M. robertsii* conidia, oral administration of avermectins and the combined treatment. Selective media for *Enterobacteriaceae* (Endo agar), *Serratia* (Serratia differential medium), *Enterococcus* and *Lactococcus* (bile esculin azide agar) were used. Different letters indicate significant differences between treatments (Dunn’s test, P < 0.05).

An analysis of CFU count on endo-agar medium indicated a significant interaction between *M. robertsii* infection and avermectin toxicosis (H_1,39_ = 4.2, P = 0.04, Fig. 4). This interaction can be explained by a significant fourfold enhancement of enterobacteria CFUs in the midgut after treatment with avermectins (Dunn’s test, P = 0.002 compared to the control) but only a twofold insignificant increase of the CFUs after the fungal and combined treatments (P > 0.12 compared to the control). Thus, fungal infection inhibited the proliferation of enterobacteria under the development of avermectin toxicosis. The same pattern was demonstrated for the CFUs on the Serratia differential medium. The factor interaction between infection and toxicosis was also significant (H_1,39_ = 8.5, P = 0.003, Fig. 4) due to a stronger elevation of the CFU count under avermectin toxicosis, although this effect was less pronounced under fungal infection and the combined treatment with fungus and avermectins. The count of CFUs on bile esculin azide agar (a medium employed for the differentiation of Firmicutes) did not exhibit any significant effects or differences between treatments.

### Expression of immunity-, stress- and ROS-related genes

**Figure 5:**
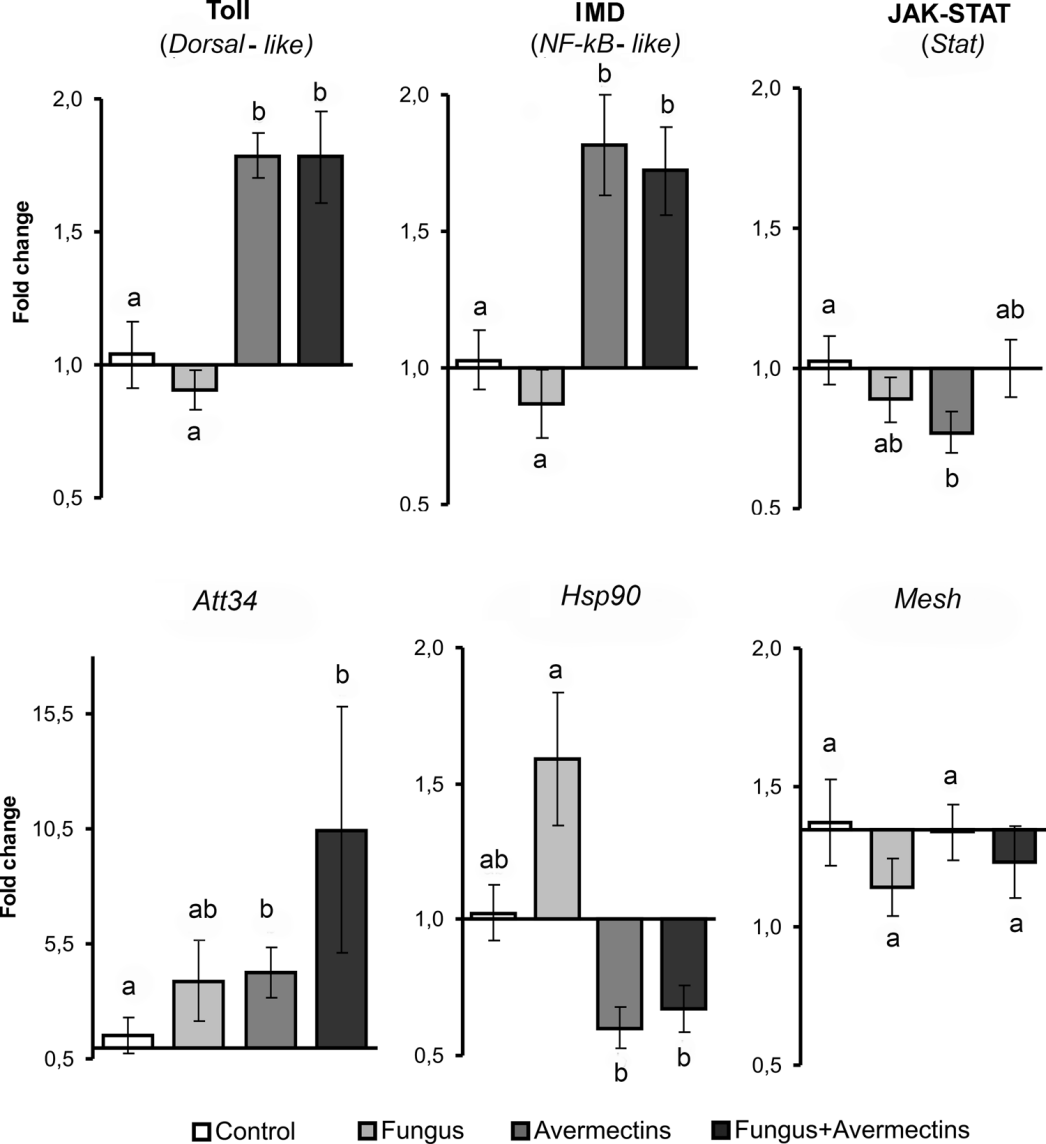
Alterations in the expression of transcriptional factors of Toll, IMD and JAK-STAT immune-signaling pathways, antimicrobial peptide attacin 34 (*Att34*), heat-shock protein (*Hsp*90) and DUOX regulator (*Mesh*) in the midgut of Colorado potato beetle larvae at 48 h after topical treatment with *M. robertsii* conidia, oral administration of avermectins and the combined treatment. Data were normalized based on the expression of two reference genes, *Rp4* and *Arf19*. Different letters indicate significant differences between treatments (Dunn’s test, P < 0.05).

We determined the upregulation of the transcription factors *Dorsal-like* and *NF-kB-like* of the Toll and IMD pathways in the midguts under the influence of avermectin toxicosis (H_1,23_ > 14.3, P < 0.0005, Fig. 5). The treatment with avermectins alone and the combination treatment (avermectins + fungus) led to 1.7- to 1.8-fold increases in the expression of these genes, respectively (Dunn’s test, P < 0.02 compared to the control). Fungal infection alone caused an insignificant 1.2-fold downregulation of *Dorsal-like* and *NF-kB-like* expression (P > 0.58 compared to the control). The transcription factor *Stat* of the JAK-STAT pathway exhibited a different pattern of expression. *Stat* was significantly downregulated after treatment with avermectins alone (Dunn’s test, P = 0.02 compared to the control). However, the combined treatment and fungal infection alone did not result in changes in *Stat* expression compared with the control. The fungus × avermectins interaction was determined to be significant (H_1,23_ = 5.5, P = 0.02). Thus, fungal infection prevented the downregulation of *Stat* caused by avermectins.

The expression of the AMP Attacin 34 (*Att34*) significantly increased after treatment with avermectins (H_1,23_ = 6.3, P = 0.01) and insignificantly increased with fungal infection (H_1,23_ = 3.1, P = 0.08, Fig. 5). Interestingly, single treatments upregulated *Att34* by 2.6–2.7-fold (Dunn’s test, P = 0.07 and P = 0.05 for mycosis and avermectin toxicosis, respectively), whereas the combined treatment upregulated this gene by sevenfold (P = 0.02 compared to the control). *Hsp90* was significantly downregulated under the influence of avermectins (H_1,23_ = 12.2, P = 0.0005) and was insignificantly upregulated after treatment with the fungus alone (P = 0.16 compared to the control); however, a significant effect of the interaction between factors on *Hsp90* expression was not observed (H_1,23_ = 0.5, P = 0.5). Expression of *Mesh* did not exhibit any significant effects, although a trend of decreased expression was observed under the influence of mycosis (H_1,23_ = 2.3, P = 0.13).

### ROS production

**Figure 6:**
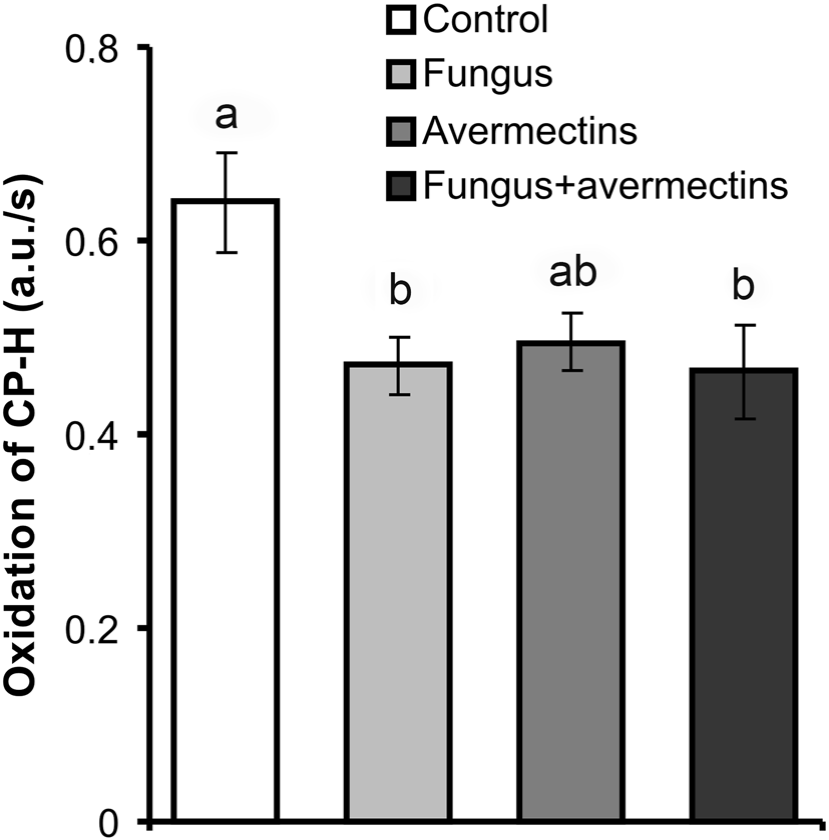
ROS production in the midgut tissue of Colorado potato beetle larvae at 48 h after topical treatment with *M. robertsii* conidia, oral administration of avermectins and the combined treatment. Different letters indicate significant differences between treatments (Tukey's test, P < 0.05).

Analysis of ROS production using electron paramagnetic resonance (EPR) spectroscopy demonstrated that *M. robertsii* infection significantly reduced the rate of ROS generation in Colorado potato beetle midguts (effect of fungus: F_1,34_ = 5.6, P = 0.02, Fig. 6). In both the fungal and combined treatments, ROS production decreased by 1.4-fold (Tukey’s test, P < 0.037 compared to the control). After treatment with avermectins, ROS production was also decreased, but the effect was not significant (F_1,34_ = 2.7, P = 0.08). The combined treatment did not lead to additional suppression of ROS production compared to individual treatments (Tukey’s test, P > 0.96). Positive correlations between the level of ROS production and diversity of the gut microbiota, especially based on the OTU count and Chao-1 index, were demonstrated (r = 0.98, n = 4, P = 0.03 and r = 0.98, n = 4, P = 0.02, respectively). Moreover, the level of ROS production was positively correlated with *Mesh* expression (r = 0.80, n = 4), although the correlation was not significant (P = 0.20).

### Detoxification enzymes

**Figure 7:**
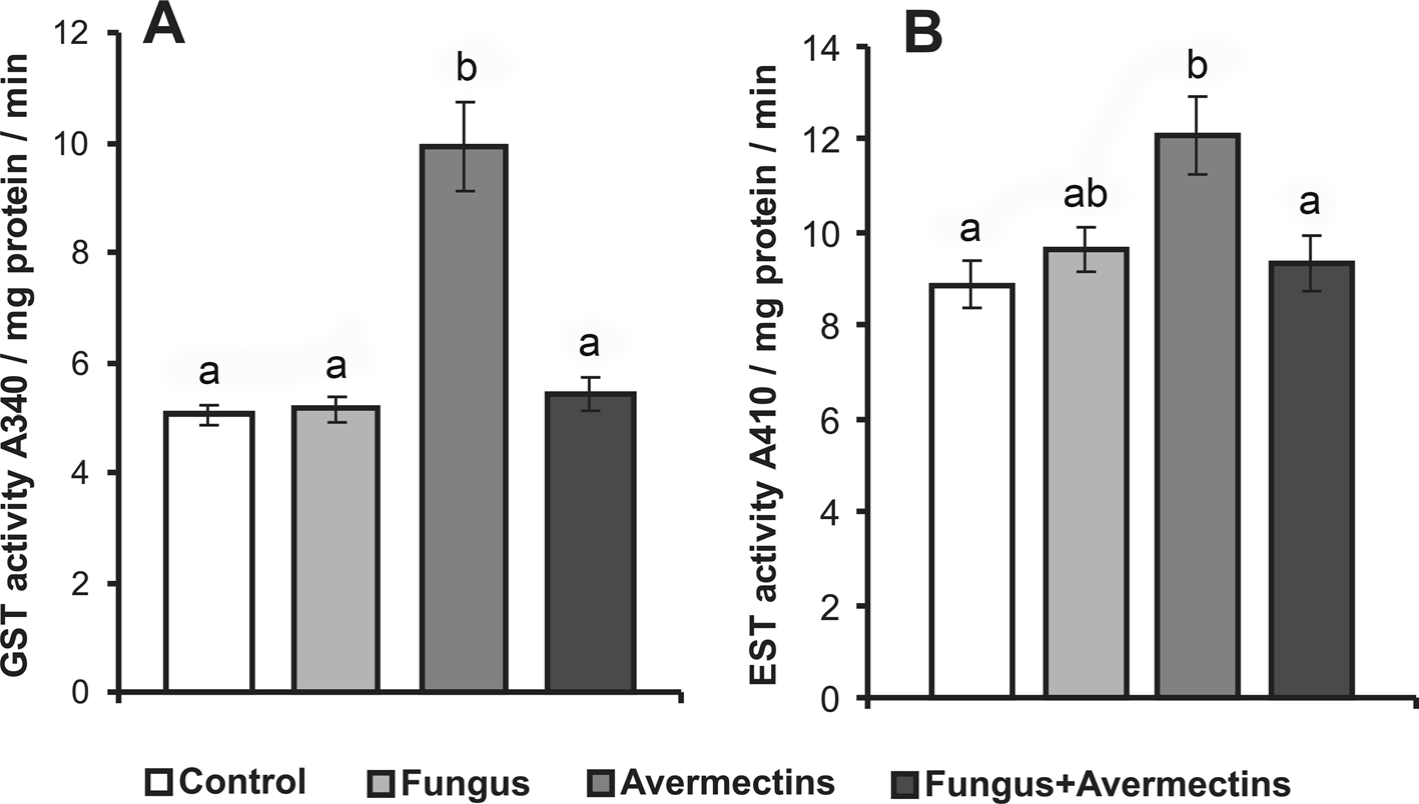
Changes in GST (**A**) and EST (**B**) activity in the midgut of Colorado potato beetle larvae at 48 h after topical treatment with *M. robertsii* conidia, oral administration of avermectins and the combined treatment. Different letters indicate significant differences between treatments (Tukey's test, P < 0.05).

We demonstrated significant factor interactions in the activities of GST and EST in the Colorado potato beetle midgut after treatment with fungus and avermectins (GST—F_1,71_ = 26.4, P < 0.001, EST—F_1,70_ = 7.4, P = 0.001, Fig. 7). Avermectin treatment alone led to a twofold increase in the GST level compared with the control (Tukey’s test, P < 0.0005); however, GST activity did not change after fungal infection alone or combined treatment with fungus and avermectins (P > 0.92 compared to the control). A similar pattern was demonstrated for EST activity. Avermectins caused a 1.4-fold enhancement in EST activity (P = 0.006, compared to the control), although such a change was not observed after fungal infection alone or the combined treatment (P > 0.95, compared to the control). Thus, fungal infection inhibited the upregulation of detoxification enzymes caused by avermectins.

### Combined effect of *Serratia* isolates and *M. robertsii* on larvae mortality

Since we observed an increase in the proliferation of the opportunistic pathogen *Serratia* under both mycosis and avermectin toxicosis (Fig. 4), we identified *Serratia* isolates and analyzed the larvae mortality dynamics after orally administering these bacteria and topical infection with *M. robertsii*. An analysis of 16S rRNA gene sequencing identified isolates belonging to *S. marcescens, S. nematodiphila* and *S. quinivorans* (Appendix 2, Table S1)*.* Weak effects on the mortality dynamics were observed between *M. robertsii* and *Serratia* isolates (Appendix 2, Fig. S1). In particular, *S. marcescens* demonstrated slow synergy (12%) only on day 5 after treatment (χ^2^ = 9.2, df = 1, P < 0.01). An analogous weak effect was detected between *M. robertsii* and *S. nematodiphila.* No synergy was observed between *M. robertsii* and *S. quinivorans*. The total mortality at day 10 after treatment for all combinations and fungal infections alone was 76–81%. It should be noted that individual administration of *S. liquefaciens* and *S. nematodiphila* led to a 19–23% increase in mortality compared to the control, and the differences were significant (log rank test: χ^2^ > 10.72, df = 1, P < 0.001). Individual administration of *S. marcescens* did not cause mortality of larvae (χ^2^ = 1.31, df = 1, P > 0.05, compared to the control).

## Discussion

This work demonstrated dramatic changes in the Colorado potato beetle midgut bacterial community after treatment with avermectins, as indicated by a reduction in diversity, proliferation of enterobacteria, and decrease in the relative abundance of *Spiroplasma*. Fungal infection also led to a decrease in the midgut microbiota diversity; however, changes in the community structure were less pronounced and were largely insignificant. Importantly, under the combined treatment (*M. robertsii* + avermectins), fungal infection inhibited enterobacterial proliferation and impeded the activation of the detoxification system in the midgut caused by avermectins. Thus, fungal infection may modulate the physiological response of insects to toxicosis caused by the insecticide in the midgut.

Overall, the structure of the Colorado potato beetle gut microbiota was consistent with earlier published data^3,42–45^, including data for the West Siberian population of the beetle, where Enterobacteriaceae and *Spiroplasma* predominated^46^. In the present study, a decrease in bacterial diversity under both avermectin toxicosis and mycosis clearly had two causes: (1) interruption of feeding, which is evidenced by the decreased abundance of chloroplast 16S DNA, and (2) elevation of enterobacteria abundance. The first finding is consistent with our previous work^40^, where we showed that the same concentrations of avermectins reduced the amount of food consumed threefold during the first day after treatment. Moreover, a trend toward a decrease in larval weight was observed after infection with the fungus *M. robertsii*^40^, which may also indicate interruption of feeding. An increase in Proteobacteria (including Enterobacteriaceae) proliferation under different pathological states has also been reported by other authors^8,9,47,48^ and may be explained by degenerative changes in the gut tissue, pH alterations, peristaltic disturbances and aeration changes. Enterobacteria are facultative anaerobes; therefore, a deceleration of peristalsis may increase their abundance. A shift in the bacterial community structure toward enterobacteria predomination was demonstrated in the wax moth gut after treatment with *H. hebetor* venom, which leads to a cessation of peristalsis^9^. A decrease in the relative abundance of obligate intracellular *S. leptinotarsa* may be explained by the elevation of enterobacteria, as well as by the destruction of midgut epithelial cells after treatment with avermectins. Damage to the gut tissue by avermectins was demonstrated for different insects, especially for mosquitoes^19^ and Colorado potato beetles (Kryukova, O. Polenogova, unpublished).

In a previous work^46^, we observed slight changes in the structure of the bacterial community and total bacterial load during the development of prolonged mycoses caused by different strains of *M. robertsii*, which is consistent with the results of the present study. However, in the present work, we documented an increase in the relative abundance and CFU count of *Serratia* during fungal infection, as well as during avermectin toxicosis. Previous studies showed that the oral administration of enterobacteria (*Serratia, Erwinia,* and *Enterobacter*) that proliferate in the gut under mycoses and other pathologies caused different insect taxa to be more susceptible to fungi^7–9^. In this study, oral administration of different *Serratia* cultures to Colorado potato beetle larvae had a weak effect on mycosis development (Fig. S1), although some *Serratia* cultures demonstrated a degree of virulence.

With fungal infection, we observed a trend of decreasing *Mesh* expression, which regulates the activity of the DUOX system. Moreover, fungal infection alone or in combination with avermectins led to a significant decrease in ROS production in the midgut. An elevation of bacterial load, especially *Serratia*, was likely caused by a reduction in ROS generation. This effect has been reported by Wei et al. regarding studies on *Anopheles*^7^. The authors suggested that a decrease in ROS production in the gut after topical treatment with *B. bassiana* was caused by fungal toxins, and the effect of oosporeins on the DUOX system was highlighted^7^. *Metarhizium* fungi produce the major metabolite destruxins that disrupt the circulation of Ca^2+^ and K^+^ ions in cells of different organs, including the gut, as shown in ex vivo tests^49^. However, at the initial stages of *Metarhizium* infection (48 h after treatment), destruxins were detected in negligible concentrations in insects^50^. The decrease in ROS production in the midgut was likely caused by prioritization of the immune response between the integuments and midgut. Insignificant effects of fungal infection on gene expression in the IMD and Toll and JAK-STAT pathways in Colorado potato beetle midgut may have been caused by the initial stage of mycosis, when the main action of fungal metabolites is the hydrolysis of integuments. Alterations in the expression of the genes in the gut were primarily identified at the latter stages of mycoses, as shown for mosquitoes^27^.

Avermectin toxicosis did not lead to changes in *Mesh* expression, and decreased ROS generation was only observed as a trend (P = 0.08). However, avermectin treatment led to a stronger perturbation of the bacterial community and the strongest increase in the bacterial load compared to fungal infection. Clearly, these changes were caused by pathological processes directly in the gut. Upregulation of transcriptional factors in the Toll and IMD pathways and the AMP attacin after avermectin treatment may be considered to be a response to the elevated bacterial load. Interestingly, avermectin treatment caused a weak reaction or inhibition of stress- and ROS-related systems in the gut. This insecticide affects the glutamate-gated chloride channel (GluCl) and is associated with the ɣ-aminobutyric acid (GABA) receptor, and it also disrupts intracellular Ca^2+^ homeostasis^51,52^, which leads to the failure of the ion-exchange function of cellular membranes, disruption of the receptors containing G-protein (GPCRs)^53^, and inactivation of mitogen-activated protein kinases (MAPК)^54^. It is possible that these processes block or downregulate the expression of stress- and ROS-related genes (*Mesh* and *Hsp90*), as well as the JAK-STAT immune-signaling pathway. However, the IMD and Toll pathways are activated through the PGRP or TLR (Toll-like receptor) and PGRP receptors^55^, and they continue to function. Therefore, we observed the upregulation of the *Dorsal-like* and *NF-kB-like* genes. Activation of the IMD and Toll pathways is likely to be less dependent on Ca^2+^ homeostasis.

It should be noted that similar patterns in gene expression in insect midguts were observed after treatment with other toxicants. In particular, envenomation of the wax moth *Galleria mellonella* by the parasitoid *H. hebetor* led to the downregulation of stress- and ROS-related genes, which was followed by the strong upregulation of AMP genes^9^. In cultured cells of *Mamestra brassicae,* induction of *Hsp90*, *Hsp70*, *Hsp20.7*, and *Hsp19.7* was caused by halogenated pyrrole chlorfenapyr but not by other insecticides from different groups, such as pyrethroids, insect growth regulators, organophosphates, carbamates and trithians^56^. In contrast, Nazir et al.^57^ and Yoshimi et al.^58^ demonstrated significant induction of *Hsp*70 expression in guts of *D. melanogaster* and in whole bodies of *Chironomus yoshimatsui* after treatment with organophosphate pesticides and pyrethroids. Chen et al. ^59^ showed that neonicotinoid imidacloprid caused insignificant upregulation of *Hsp*70 in whole bodies of Colorado potato beetle larvae under optimal temperature (25 °C) and slight downregulation in response to the insecticide under high temperature (43 °C). Thus, the change in expression of HSPs may depend on the insecticide group, insect taxa and environment. However, it appears that neurotoxic insecticides do not cause significant change in expression of HSPs in Colorado potato beetles.

Importantly, the combined treatment (fungus + avermectins) did not lead to an additional decrease in microbiota diversity and increase in the bacterial load compared to the single treatments. In contrast, the fungus inhibited the elevation of the enterobacteria load with the combined treatment (Fig. 4). Joint treatment with fungus and avermectins did not lead to any additional effects on ROS generation and *Hsp*90, *Dorsal-like* and *NF-kB-like* gene expression levels, although a stronger enhancement of *Att*34 gene expression was observed. Interestingly, fungal infection prevented the downregulation of *Stat* caused by avermectins (Fig. 5). It is likely that fungal infection may regulate the proliferation of bacteria after treatment with additional factors, such as insecticides. Regulation of insect bacterial communities by entomopathogenic fungi was observed in the final stages of mycoses owing to fungal secondary metabolites^60^, although it was not observed at the initial stages. We surmise that the regulation of bacterial load by fungi after combined treatment may be mediated by host immune reactions, particularly the regulation of the *Att*34 and *Stat* genes.

The JAK-STAT pathway is activated by cytokines produced after damage to the gut epithelium, and this pathway participates in AMP production, as shown in *Drosophila*^26,29^. Upregulation of JAK-STAT pathway genes was observed in the gut of *Bombyx mori* after infection with different gram-negative and gram-positive bacteria^61^. In addition, JAK-STAT is involved in the insect antifungal response^27,28^. A decrease in the bacterial load under the combined action of fungus and avermectins (compared to action of avermectins alone) may have been caused by preventing JAK-STAT downregulation by fungus. We hypothesize that this effect may be caused by a systemic response of larvae to fungal infection, as demonstrated for mosquitoes by Ramirez et al. ^27^. To generate such evidence, it is necessary to analyze JAK-STAT gene expression in different tissues (e.g., cuticle, fat body, and hemocytes) after fungal infection of Colorado potato beetle, as well as investigating the effects of JAK-STAT silencing on bacterial communities.

In this study, considering the processes that occur in the midgut, we did not find direct confirmation that avermectins may promote fungal infection, as indicated in studies on the hemolymph and cuticle of the beetle^39^. However, we observed another effect in which fungal infection impeded the detoxification response to avermectins. In particular, *M. robertsii* infection prevented the activation of GST and EST in the midgut in response to avermectin treatment. EST and GST are involved in resistance to avermectins. Increased GST and EST levels were observed in arthropod lines that demonstrated resistance to this insecticide^62–64^, and inhibition of the detoxification enzymes led to an increase in susceptibility to abamectin^51,65–67^. In particular, direct silencing of GSTz2 expression increased the susceptibility of the fruit fly *Bactrocera dorsalis* to abamectin^67^. In Colorado potato beetle larvae, inhibition of esterases by S,S,S-tributyl phosphorotrithioate led to a fivefold increase in susceptibility to abamectin, and inhibition of GST by diethyl maleate caused a twofold increase in susceptibility to this insecticide^68^. Moreover, the inhibition of esterase activity may increase insect susceptibility to bacterial toxins^35^. Thus, blocking detoxification enzyme activation in Colorado potato beetle gut by the fungus may lead to increased toxic effects of avermectins, which may lead to general physiological consequences, such as a delay in development, weakening of cellular immunity and changes in the thickness and biochemical properties of integuments^39,40,69^. These disruptions are crucial to the susceptibility of Colorado potato beetle to *Metarhizium* and *Beauveria*^69,70^. It is likely that both direct and inverse effects between fungal infection and avermectins may occur in the investigated system and may lead to the formation of a "vicious circle" in which avermectins promote fungal infection (in the cuticle and hemolymph), and this infection increases the toxic effect of avermectins (in the gut). These interactions may cause a synergistic effect between the fungus and the avermectins. It should be noted that similar alterations of the detoxification enzyme activities under the combined action of *M. robertsii* and avermectins were established on mosquito *Aedes aegypti* larvae at the initial stages of mycosis and toxicosis^71^, which is in keeping with our hypothesis.

In conclusion, this study is the first to show alterations in the gut immune reactions and bacterial community of Colorado potato beetle after treatment with fungal infection, insecticide and both. We demonstrated that a decrease in ROS production and elevation of *Serratia* proliferation occurred in the midgut of beetle larvae after topical treatment with fungus, which is consistent with the results obtained for mosquitoes^7^ and supports the hypothesis that immune reactions are prioritized between the integuments and gut under the development of fungal infections. Avermectin toxicosis led to a significant shift in bacterial communities toward enterobacterial prevalence and activation of the Toll and IMD immune-signaling pathways. However, downregulation of stress-regulated genes (*Hsp*90) and the JAK-STAT pathway was observed after treatment with this insecticide, which may be attributable to the disruption of receptors containing G-protein. Significant interactions between fungal infection and avermectin toxicosis were demonstrated for the gut immune responses and bacterial load. In particular, fungal infection impeded the elevation of enterobacteria, downregulation of STAT and activation of detoxification enzymes caused by avermectins. Blocking the detoxification response by fungi may be one of the mechanisms governing the synergy between *M. robertsii* and avermectins. Further research may investigate the temporal-spatial distribution of immune reactions in Colorado potato beetles under mycoses and toxicoses.

## Methods

### Fungi, insects, and insecticide

Colorado potato beetle larvae were collected from private potato fields in the steppe zone of Western Siberia (53° 44′ N, 78° 02′ E) in July 2018. Strain P-72 *M. robertsii *(GenBank no. KP172147) from the microorganism collection of the Institute of Systematics and Ecology of Animals, Siberian Branch of Russian Academy of Science (SB RAS) was used in the experiments. Conidia were grown on autoclaved millet as described previously^69^. For the infection, conidia were suspended in a water-Tween 20 solution (0.03%) to a final concentration 10^6^ conidia/mL. The concentration was determined using a Neubauer hemocytometer. The viability of the conidia was checked by plating on Saburoad dextrose agar. The germination of the conidia was > 95%. The industrial product Actarophyt 0.2% (Enzyme, Vinnytsia, Ukraine) with a complex of natural avermectins produced by *Streptomyces avermitilis* (http://enzim.biz) was used. Actarophyt dissolved in distilled water to 0.0054% (half-lethal concentration) was used for the treatment. Selected concentrations of conidia and avermectins enable synergy between these agents^39,40^^.^

### Procedure of treatments and bioassay

Larvae (4–6 h after molting at the fourth instar) were infected with fungus by being dipped for 15 s into a water-Tween suspension of conidia. Control larvae were treated with a conidia-free water-Tween solution. After inoculation, the larvae were immediately placed on *Solanum tuberosum* leaves treated with either the avermectin solution or distilled water. The following four treatments were set up: Control, Fungus, Avermectins and Fungus + Avermectins. Larvae were maintained in 300 mL ventilated plastic containers (10 larvae in each) with potato foliage (6 g per container daily). To prevent plant desiccation, leaf footstalks were inserted in 2.5 mL tubes that were plugged with damp cotton. Containers were exposed at 25–26 °C, 25–30% RH and 14:10 h photoperiod. Insect mortality was registered daily for 10 days. At least 60 larvae of each treatment were used for the bioassay.

### Midgut bacterial communities

At 48 h after treatment, the larvae midguts with contents were isolated and frozen in liquid nitrogen (5 midguts per sample) and stored at − 80 °C. Total DNA was extracted using the DNeasy PowerSoil Kit (QIAGEN). Bead beating was performed using a TissueLyser II (QIAGEN) for 10 min at 30 Hz. The V3-V4 region of the 16S rRNA genes was amplified with the primer pair 343F and 806R as described early^72^. The 16S libraries were sequenced with 2 × 300 bp paired-ends reagents on the MiSeq platform (Illumina) at the SB RAS Genomics Core Facility (Institute of Chemical Biology and Fundamental Medicine SB RAS, Novosibirsk, Russia).

Raw sequences were analyzed with the UPARSE pipeline^73^ using USEARCH v11.0.667. The UPARSE pipeline included paired read merging, read quality filtering, length trimming, identical read merging (dereplication), discarding singleton reads, removing chimeras and OTU clustering using the UPARSE algorithm^74^. The OTU sequences were assigned a taxonomy using the SINTAX^75^ and 16S RDP training set v16 as a reference^76^. The OTUs related to chloroplast 16S rDNA were removed from the final data set and analyzed separately.

The final data set included 507,752 reads (31,734 ± 2440 per sample) (see Appendix 1). Rarefaction and extrapolated curves were generated using the “iNEXT” package^77^ and showed trends approaching the saturation plateau, which indicated that enough reads were included (Appendix 2, Fig. S2). Alpha diversity metrics were calculated in Usearch.

### Analysis of CFU count

Colorado potato beetle midguts with contents (1 sample = 3 midguts) were homogenized and suspended in sterile 150 mM NaCl solution. Aliquots of 100 µL suspension (5 × 10^–2^–5 × 10^–5^) were inoculated onto the surface of selective media. The CFUs of *Lactococcus* were determined by plating onto bile esculin azide agar M493 (HiMedia, Mumbai, India); those of Enterobacteriaceae (excluding *Serratia*) were determined by plating onto endo agar M029; and those of *Serratia* were determined by plating onto Serratia differential medium M1288 (same manufacturer). Petri dishes with bacteria were incubated at 26 °C for 48 h. Then, the bacterial colonies were counted based on the morphological characteristics. At least 5 replicates for each treatment were used for analysis.

### qPCR analysis of immunity-, stress- and ROS-related genes

Colorado potato beetle midguts were isolated at 48 h after treatment, dissected in PBS and cleaned and washed to remove the contents of the gut lumen. All procedures were performed according to previously described techniques^9^. Briefly, five midguts were pooled in one sample, and six biological replicates per treatment were used in the analysis. Total RNA was extracted by QIAzol Lysis Reagent for DNA, RNA and protein isolation (QIAGEN) according to the manufacturer’s instructions. qPCR analysis was performed in three technical replicates under the following conditions: 95 °C for 3 min followed by 40 cycles of 94 °C for 15 s and annealing and elongation at 62 °C for 30 s; and generation of melting curves (70–90 °C). Two genes of *Leptinotarsa decemlineata* were used as reference genes: 60S ribosomal protein L4 (*Rp4*) and ADF-ribosylation factor-like protein 1 (*Arf1*). These genes and primer sequences were from the work of Shi et al. ^78^ and were more stable in our investigated system. The relative expression of six genes of interest (*Dorsal-like*, *NF-kB-like, Stat, Att34, Hsp90,* and *Mesh*) was assayed. Primer sequences are provided in Appendix 2, Table S2. Primer properties were identified by IDT OligoAnalyzer 3.1 (http://eu.idtdna.com/calc/analyzer).

### EPR analysis of ROS production

Spin trapping using 1-hydroxy-3-carboxy-pyrrolidine (CP-H) has been used to measure the rate of ROS formation^79^. CP-H rapidly reacts with oxygen-centered free radicals, including superoxide, and some molecular oxidants to generate stable nitroxide that can be detected by EPR. In contrast, the reaction with oxygen, hydrogen peroxide and organic peroxides is very slow^80^.

ROS measurement was performed as previously described^79^ with modifications to the sample preparation. Colorado potato beetle midguts were isolated at 48 h after treatment, dissected in PBS and cleaned and washed to remove the contents of the gut lumen. The sample contained 5 guts homogenized in 50 μL sodium phosphate buffer (50 mM with 0.1 mM DTPA, pH 7.2), and 1 mM CP-H was placed in a 50 μL glass capillary tube for EPR measurement. Ten samples were analyzed in each treatment.

### Detoxification enzymes

To measure GST and EST activity, we utilized the technique of Habig et al.^81^ and Prabhakaran and Kamble^82^ with minor modifications. In brief, one sample included 2 guts that were homogenized in 100 μL sodium phosphate buffer (0.1 M pH 7.2) (PBS) with phenylthiourea (4 mg/mL) and centrifuged at 10,000*g* for 10 min. The GST activity was estimated by measuring the generation of 5-(2,4-dinitrophenyl)-glutathione at a wavelength of 340 nm. The EST level was estimated according to p-nitrophenyl acetate hydrolysis at 410 nm. The protein concentration was estimated using the Bradford method^83^. Bovine serum albumin was used for the calibration curve. At least 15 samples were analyzed in each treatment.

### *Serratia* identification and their influence on susceptibility to fungus

Representative colonies from Serratia differential medium were selected and passaged three times on tryptose agar medium. Bacterial strains were identified as previously described^9^ by sequencing a 1308-bp fragment of the 16S rRNA gene using primers 27F 5′-AGAGTTTGATCMTGGCTCAG-3′ and 1492R 5′-CCCTACGGTTACCTTGTTACGACTT-3. The obtained 16S rRNA gene sequences were compared against GenBank-available sequences from the type material (Appendix 2, Table S1).

For the bioassay, potato leaves were dipped in suspensions of *Serratia* isolates (10^9^ propagules/mL) or distilled water (controls) and dried at 30 m under 25 °C. Larvae were dipped in the suspension of *M. robertsii* (10^6^ conidia/mL) or conidia-free water-Tween solution and immediately placed on treated or untreated potato leaves. The following four treatments were used: Control, *Serratia*, Fungus, and *Serratia* + Fungus. Larvae were maintained as described above. Mortality was measured for 10 days. At least 40 larvae (1 replicate = 10 larvae) were used in each treatment.

### Statistical analyses

Data analyses were conducted using PAST 3^84^, STATISTICA 8 (StatSoft Inc., USA), SigmaStat 3.1 (Systat Software Inc., USA), and AtteStat 12.5^85^. Data were checked for a normal distribution using the Shapiro–Wilk W test. Normally distributed data were analyzed by two-way ANOVA, followed by Tukey’s post-hoc test. For abnormally distributed data, we used a nonparametric equivalent of the two-way ANOVA Scheirer–Ray–Hare test^86^, followed by Dunnʼs post-test. The synergistic effects in mortality were detected by comparing the expected and observed mortality as described by Robertson and Preisler^87^. In addition, the log-rank test was applied for estimating differences in the mortality dynamics. Data on the plots are presented as the arithmetic means and standard errors.

## Supplementary Information

Supplementary Information 1.

Supplementary Information 2.

## Data Availability

The MiSeq data were deposited in GenBank under the study accession number PRJNA613617. The sequences of the 16S rRNA genes of the *Serratia* strains were deposited in the GenBank database under accession numbers MT256278, MT256279, MT256306 and MT256307. Experimental data are presented in Appendix 1.
