# Supplementary material for: Fungus Metarhizium robertsii and neurotoxic insecticide affect gut immunity and microbiota in Colorado potato beetles
Source: Sci Rep. 2021 Jan 14;11:1299. doi: 10.1038/s41598-020-80565-x (PMC7809261; doi:10.1038/s41598-020-80565-x)
Supplement: Supplementary file 2 — Supplementary Information 2. [file 41598_2020_80565_MOESM2_ESM.pdf]

APPENDIX B

Fungus *Metarhizium robertsii* and neurotoxic insecticide affect the gut immunity and microbiota in Colorado potato beetle

Vadim Yu. Kryukov, Ulyana Rotskaya, Olga Yaroslavtseva, Olga Polenogova, Natalia Kryukova, Yuriy Akhanaev, Anton Krivopalov, Tatyana Alikina, Yana Vorontsova, Irina Slepneva, Marsel Kabilov, Viktor V. Glupov

Table S1. Putative identification of 16S rRNA gene sequences of isolates of bacteria of the genus Serratia from the gut of the Colorado potato beetle using the BLAST algorithm compared to similar sequences obtained from GenBank

Isolated	GenBank accession numbers	Nearest match	GenBank accession numbers	Identity (%)
0218	MT256306	*Serratia nematodiphila*	NR_044385.1	100
3318	MT256278	*Serratia quinivorans*	NR_037112.1	99.77
10918	MT256307	*Serratia marcescens*	NR_114043.1	99.86
5618	MT256279	*Serratia quinivorans*	NR_037112.1	99.78

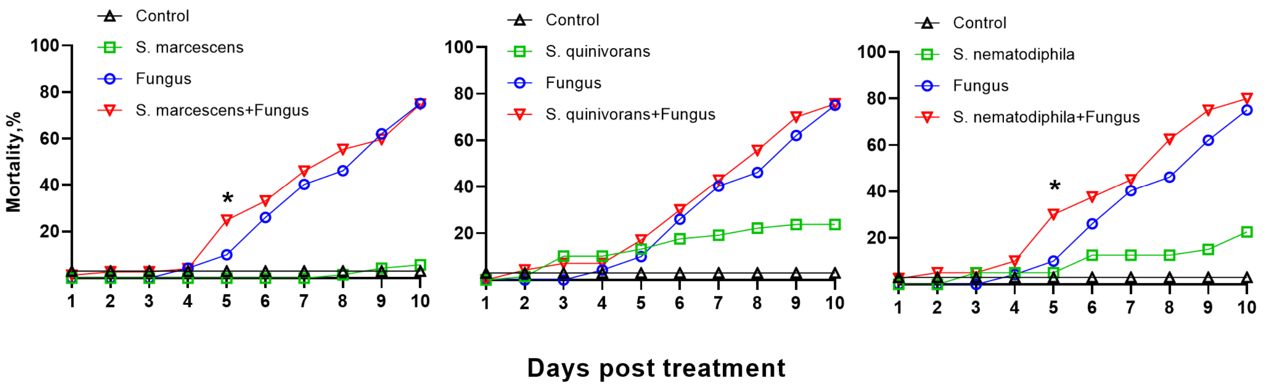

Fig. S1. Mortality dynamics of Colorado potato beetle larvae after topical treatment with *M. robertsii*, oral administration of *Serratia* species and the combined treatment; * - synergistic effect ($\chi^2 > 3.8$, df = 1, P < 0.05).

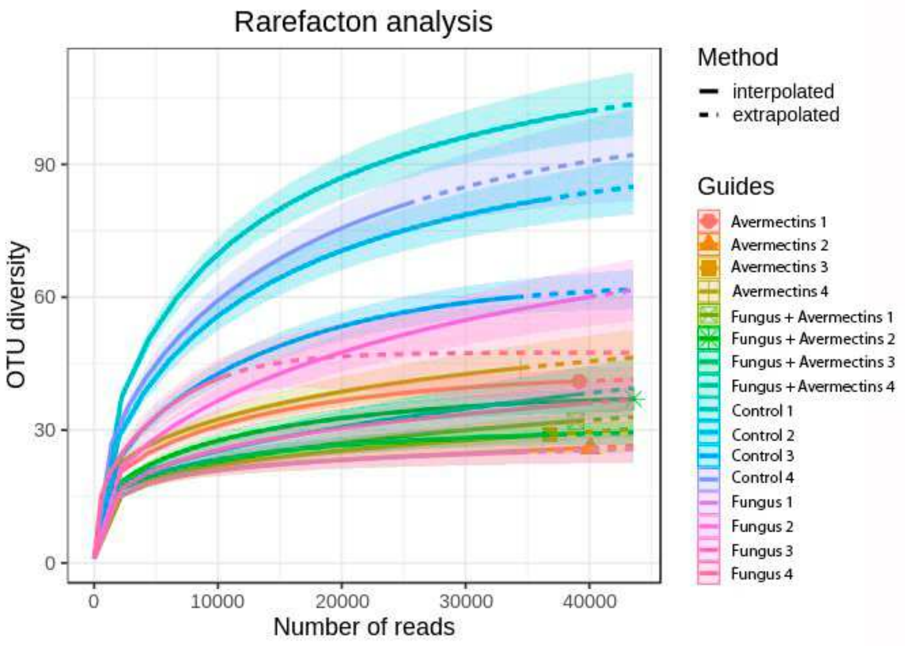

Fig. S2. Rarefaction curves of the OTU number for each sample.

Table S2. List and description of genes and primers sequences used in the qPCR.

Gene Name	NCBI GenBank Accession number	Gene symbol	Primer sequence (5' – 3')	Product size (bp)	PCR efficiency (±SD)	Tm in qPCR, °C	Primers source
60S ribosomal protein L4	XM_023165859.1	*Rp4*	For GAAACGAGCATTGCCCTTCC<br>Rev TCGCTGACACTGTAGGGTTG	119	1,99±0,03	62	[a] modified
ADF-ribosylation factor-like protein 1	XM_023169879.1	*ARF1*	For CGGTGCTGGTAAAACGACAATATT<br>Rev TGACCTCCCAAATCCCAAACTT	135	0,97±0,04	62	[a] modified
DorsalDif-like protein	XM_023158121.1	*Dorsal-like*	For TGTGCGAAAAGGTGGCTAAAG<br>Rev ACTTGGGAGGGTTGGAAGTC	94	1,93±0,07	62	[b] ISaEA
NF-kappaB-like protein	XM_023174540.1	*NF-kB-like*	For AAGCAGCGGTTTGATTCGTTC<br>Rev AACTCGTCCAAGTTCTCCAGG	120	1,97±0,03	62	[b] ISaEA
Signal transducer and activator of transcription protein	XM_023165198.1	*Stat*	For AGGAGCAGAACACAGGGTAC<br>Rev TTTGCCTGGGAATTCTGTTGAC	140	1,93± 0,08	62	[b] ISaEA
Attacin_C domain contained protein	XM_023168834.1	*Att34*	For TGAGAACTCCACAAAATATTCACTTCG<br>Rev CTAAGGGTATGGCAGCAACAAC	98	1,95±0,04	62	[b] ISaEA
Heat shock protein 90	KC556802.1	*Hsp90*	For GGGTGTAGTCGACTCTGAAGAC<br>Rev AGAGCTCCTCAAACAGTTCCAA	125	1,89±0,01	62	[b] ISaEA
Gut membrane-associated protein Mesh	XM_023158552.1	*Mesh*[a]	For CGCAATGGTTCAACGATATGGT<br>Rev GCTTGGTACGCGACACAGT	124	1,98±0,01	62	[b] ISaEA

[a] Shi X.-Q. et al., Validation of reference genes for expression analysis by quantitative real-time PCR in *Leptinotarsa decemlineata*(Say) Shiet al. BMC Research Notes 2013, doi: 10.1186/1756-0500-6-93

[b] Primers were designed by Rotskaya U. in programs Primer-BLAST https://www.ncbi.nlm.nih.gov/tools/primer-blast/, IDT OligoAnalyser Tool and IDT UNAFold Tool https://eu.idtdna.com .
